# Key clinical research priorities for the pediatric community during the COVID-19 pandemic

**DOI:** 10.1038/s41390-020-0962-y

**Published:** 2020-05-15

**Authors:** Gary J. Noel, Jonathan M. Davis, Octavio Ramilo, John S. Bradley, Edward Connor

**Affiliations:** 1 Institute for Advanced Clinical Trials for Children, Rockville, MD USA; 2 Weill-Cornell Medical College, New York, NY USA; 3 The Floating Hospital for Children at Tufts Medical Center and the Tufts Clinical and Translational Science Institute, Tufts University School of Medicine, Boston, MA USA; 4 Ohio State University College of Medicine, Nationwide Children’s Hospital, Columbus, OH USA; 5 Rady Children’s Hospital-San Diego, University of California San Diego School of Medicine, San Diego, CA USA; 6 George Washington School of Medicine, Washington, DC USA

As of May 1, the Johns Hopkins Coronavirus Research Center is reporting that 3.127 million cases of coronavirus disease 19 (COVID-19) and 213,791 deaths related to these infections have occurred worldwide (https://coronavirus.jhu.edu/). As the pandemic evolves, information about how the virus has impacted the health of children is being steadily reported. In early April, the CDC calculated that in the US approximately 1.7% (2572 of 149,082 for which age was reported) of COVID-19 cases had occurred in children <18 years of age.^1^ Seventy-three percent of these cases were associated with fever, cough, and/or shortness of breath and 5.7% were hospitalized, compared to 93% and 10% respectively in adults. The median age of cases in children was 11 years (range 0−17 years) with 33% reported in children 15−17 years of age and 15% in children <1 year of age. In this dataset, 23% of children had underlying medical conditions which were present in 77% of hospitalized children and all six children admitted to the ICU. The experience in the first months of the pandemic suggests that although susceptibility to infection may be similar in children and adults, the risk for severe disease appears to be lower in children.^1–3^ Nevertheless, serious COVID-19 has been reported in children, including neonates/young infants and those with underlying medical conditions.^2,4^

Given the need to urgently respond to the pandemic, the immediate priority of pediatricians has been to provide health care to those most severely ill, even if this means helping care for adult and elderly patients. However, as the pandemic matures, it is also very important for the pediatric clinical research community to start immediately identifying key knowledge gaps and to fill those gaps to best address the needs of children. This is particularly true where information needs to be clinically applied in real time to inform clinical research and advance the field related to COVID-19 infections in children at the same pace we are likely to see for adults. For children, several key issues require urgent preparation and action (Table 1).

**Table 1:** Key areas for clinical research on COVID-19 in pediatric populations.

Pathophysiology and natural history	• Characteristics of SARS-CoV-2 infection in neonates, children and adolescents.
	• Correlates of risk, progression, recovery and protection.
	• Studies of transmission to and from children, including household transmission, particularly in the elderly.
Viral diagnosis	• Development of rapid point-of-care diagnostics for children and methods for assessment of viral load and shedding of infectious virus.
	• Large-scale testing of pediatric patients to determine rates of infection and potential coinfection with other respiratory viruses.
	• Multiyear assessments of recurrent infection in infants/children.
Detection of antibodies	• Development of rapid point-of-care tests for detection of SARS-CoV-2 Ab.
	• Methodology for detecting and quantitating neutralizing Ab.
	• Long-term studies of durability of antibody response.
Framework for antiviral development	• Knowledge base for information about candidates in development.
	• Pediatric engagement early in development for input regarding pediatric needs and optimal planning for pediatric studies.
	• Mechanism for input regarding risk: benefit assessment.
Evaluation of vaccine candidates	• Assessment of developmental effects on vaccine response.
	• Evaluation of risk for enhancing responses to vaccine candidates.
	• Pediatric engagement early in development for input regarding pediatric needs and optimal planning for pediatric studies.
	• Mechanism for input regarding risk: potential benefit.

*Understanding the pathophysiology and clinical course of COVID-19 infection in children and the impact of maternal infection during pregnancy on the neonate*: This understanding is essential for developing the best therapeutic and preventive strategies to address medical needs in neonates, infants, children and pregnant women and represents the highest research priority. To date, the preliminary observation that children are at lower risk than adults for serious COVID-19 disease has not been fully explained. Several hypotheses have been proposed including developmental differences in receptor density and distribution; more robust/effective immune response; and better end-organ reserve because of fewer comorbidities.^2^ Validating the observation that children are at lower risk for serious disease, understanding the number of asymptomatic infants and children capable of transmitting infection, and defining the underlying pathophysiology of disease in children is foundational to the search for ways to prevent and treat COVID-19. The National Institutes of Health (NIH) and other research enterprises worldwide are well positioned to address these issues in collaboration with the pediatric research community. Given the urgency, it will be important to increase efficiency by establishing a clear roadmap for addressing these issues.

*Ensuring availability of widespread, rapid point-of-care diagnostic testing*: Identification of active COVID-19 infection is important for children as well as adults. Children appear to be quite susceptible to COVID-19 infection and viral replication/shedding appears to occur in both the respiratory and gastrointestinal tracts.^2^ Identifying infected children is critical to implementing infection control practices in the community and in medical care settings. This is based on the experience with influenza and can be expected to be extremely important in public health strategies aimed at preventing infection in the most susceptible patients. More widespread availability of testing will also allow better understanding of the clinical course of infection in children and is needed to establish the risk of serious illness in infants and children. Restrictive criteria for testing (e.g. high-risk exposure history, travel, symptoms) currently included in public health guidelines have largely been driven by limited supply of testing kits and are likely to result in substantial underestimation of the number of COVID-19 cases in children. Validation of the natural history and impact of disease in children will require broadly available point-of-care testing and possible modifications of testing methods to be acceptable for use in neonates, infants, older children, and adolescents. Pediatric health-care providers are well versed in viral diagnostics and, once given better access to testing for COVID-19, can be expected to rapidly generate the data needed to construct appropriate protocols that will test investigational candidates for prevention and/or treatment.

*Conducting widespread serologic testing of children as a marker for susceptibility*: Antibody testing for COVID-19 is now being introduced for clinical use. It is critical to initiate widespread testing in children to assess the movement of the viral infection through children in communities and the impact of children on the spread of disease into adult populations, particularly the elderly. Although it is appropriate (until proven otherwise) to assume that the presence of anti-COVID-19 serum antibodies is evidence of prior infection, it has yet to be determined if this is associated with protection from new infections (https://www.idsociety.org/globalassets/idsa/public-health/covid-19/idsa-covid-19-antibodytesting-primer.pdf). In addition, the duration of any protection will need to be defined. Identification of protective immunity in children will be key for policymakers to decide about when and how to return children to daycare and schools and to ensure the relative safety of extended family caregivers (e.g. grandparents and other higher-risk individuals).

Antibody testing, including assessment of correlation between binding antibody titers measured by ELISA and neutralizing antibody titers, is needed for: (1) establishing research initiatives to validate “protection”; (2) establish the natural history of COVID-19 in children; and (3) optimal design of safe and effective vaccines. For all these reasons, antibody testing that is pediatric friendly can be point of care with rapid readout of results, and is available in resource poor as well as other settings is necessary. NIAID has recently begun a serological survey of 10,000 adults (>18 years of age) in the US with no history of COVID-19 symptomology to assess the frequency of unrecognized prior infection (https://www.nih.gov/news-events/news-releases/nih-begins-study-quantify-undetected-cases-coronavirus-infection). Parallel studies in children are needed as soon as feasible and should be designed to address the potential for some immune responses to be associated with exacerbation of subsequent infections with coronaviruses. How information about seropositivity (and presumed “protection”) is utilized to make policy decisions involves serious economic, public-health, and bioethical considerations. Ensuring that scientifically valid data are collected is the responsibility of the pediatric research community and forms the basis for making these complex decisions.

*Establishing a framework for evaluation of safety and efficacy of new therapeutic agents for COVID-19 in pediatric populations*: As the impact of COVID-19 increases, the need for treatment modalities for the highest risk populations is an urgent priority. Approaches have included re-purposing existing agents as well as developing innovative drugs and biologics.^5^ To date, multiple agents are in development and more are being introduced every day. These include immune serum and other biologic agents (including vaccines), antiviral drugs, and candidate treatments for supportive care. As these candidate therapeutics are developed and tested in adults, consideration should be given to innovative ways to ensure evaluation in children and adolescents as soon as appropriate based on risk and potential benefits. To do this, consideration of what may be needed from adult studies to allow extrapolation or simulation to be applied in children is essential. It is important for pediatricians to be at the table when adult studies are being planned to help optimize collection of standardized data elements needed in subsequent studies in children. Depending on the characteristics of a treatment candidate, investigators will need to have a good understanding of the risk of COVID-19 infection in children to ensure optimal risk/benefit deliberations. The profile of risk for children for many therapeutic agents may be very different than adults due to significant differences in pathophysiology, drug metabolism, and multiple other considerations. However, as more information becomes available, risk/benefit considerations may change substantially. The inclusion of adolescents in adult trials has been recommended in oncology and when appropriate in other drug development settings. If the pathophysiology of disease is similar in adolescents and adults, the illness in the adolescent patient being considered is severe and the risk of the new therapy is assessed to be low, adolescents should be included in adult trials. On average, the approval of innovative therapeutics for children often lags about a decade behind approval in adults. This delay is often caused by a failure to include children at very early stages and to assume that these therapies cannot be assessed in children until studies in adults are completed. While it appears so far that children represent a small proportion of the total population with serious COVID-19 disease, this should not mean that we are left without the information needed to make reasonably well-informed decisions about using new therapies in children who are critically ill. Antiviral drug development has made enormous progress over the last decades and early consideration and inclusion of children into clinical trials has resulted in a much better track record than in some other areas; a lesson clearly learned during the response to HIV infections in infants and children at the end of the last century.

*Evaluation of vaccines and other preventive measures for children*: There is intense interest in ongoing efforts to develop vaccine candidates for the prevention of COVID-19. Pediatricians have significant expertise with the process for evaluation of the safety and efficacy of vaccines and the development of recommendations for their use. Clearly, new vaccine candidates need to be evaluated in children as soon as scientifically and medically appropriate given their likely role in COVID-19 transmission. Planning for evaluation in children will require continuous monitoring of the viability of vaccine candidates and risk/benefit assessments to determine the appropriate timing for pediatric clinical trials. In addition, for some candidate vaccines, the risk of potential exacerbation of disease (e.g. seen in early trials of Respiratory Syncytial Virus—RSV vaccine candidates) will require special attention. Development of new vaccines for respiratory viruses has historically been a lengthy process as was evident in the development of influenza vaccines in children and is evident with efforts to develop a vaccine for RSV. A roadmap for the development of a COVID-19 vaccine for children should be developed based on evolving science and experience.

The main reason why children are left behind in the evaluation of new interventions is the failure to think and plan pediatric studies early in the development process. Science, commitment, funding, and risk/benefit will determine how well we as a community respond to the COVID-19 pandemic. Early consideration and planning for including children in these drug development programs is essential. In the past decade, the pediatric research infrastructures for product development in the United States, European Union, Japan and elsewhere have worked to create mechanisms for public−private global collaboration to advance product development for children. These efforts should ensure that infants, children, adolescents and pregnant women are included in the development of preventive and therapeutic approaches to stem the impact of COVID-19. This experience will not only address the specific challenges related to COVID-19, but can also define a pathway for working efficiently to address the needs of infants and children posed by pandemics we might face in the future.
